# Xanthogranulomatous cystitis: A rare clinical entity

**DOI:** 10.4103/0974-7796.68863

**Published:** 2010

**Authors:** Santosh K. Singh, Atul K. Khandelwal, Devendra S. Pawar, Rajeev Sen, Sachit Sharma

**Affiliations:** Department of Urology, Pt. B. D. Sharma Postgraduate Institute of Medical Sciences, Rohtak, Haryana, India; 1 Department of Pathology, Pt. B. D. Sharma Postgraduate Institute of Medical Sciences, Rohtak, Haryana, India

**Keywords:** Inflammatory disease, urinary bladder, xanthogranulomatous cystitis

## Abstract

Xanthogranulomatous cystitis (XC) is a rare benign disease of unknown etiology. A case of XC in a 30-year-old male is presented due to sparcity of such case report in medical literature. Patient evaluation included clinical, biochemical and radiological studies before treatment. Histological study revealed the rare diagnosis. Patient was asymptomatic at eight weeks follow-up after treatment.

## INTRODUCTION

Xanthogranulamatous changes has been reported to occur in many sites[1,2] including the colon, ovary, pancreas, salivary gland, appendix, gallbladder, endometrium, brain, and kidney.[3] However, xanthogranulomatous cystitis (XC) is a rare, benign chronic inflammatory disease of unclear etiology and was first described in 1932[4] and presentation as bladder mass on lateral wall is rarest. The case alone with its management is hence being reported in order to contribute to medical knowledge.

## CASE REPORT

**Figure 1:**
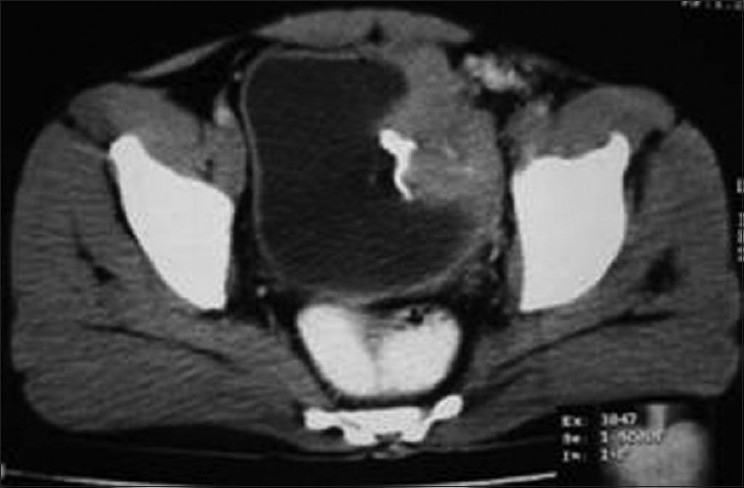
Computed tomography of the pelvis shows lobulated mass with contrast enhancement on left lateral wall of bladder

A 30-year-old male presented with sixth month history of urgency, frequency, dysuria, hematuria and lower abdominal pain. He had no significant past medical history. Physical examination was normal. Laboratory studies revealed normal hematological and biochemical profile. Urinalysis shows 10–20 red blood cells and plenty of white blood cells per high power field. Urine culture grew *E. coli* and urine cytology revealed no malignant cells. Ultrasonography showed thickened wall urinary bladder with normal capacity and large hypoechoic lesion of 60 mm×55 mm on left lateral wall of urinary bladder with good vascularity on color doppler. Contrast enhanced computed tomography (CT) demonstrated 65 mm×55 mm growth present on left lateral wall of urinary bladder. No invasion of surrounding organ noted [Figure 1].

**Figure 2:**
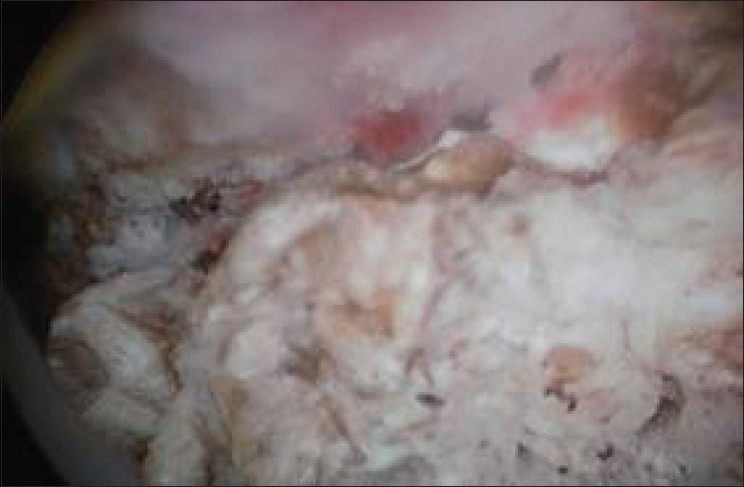
Cystoscopy reveals growth on left lateral wall of the bladder

**Figure 3:**
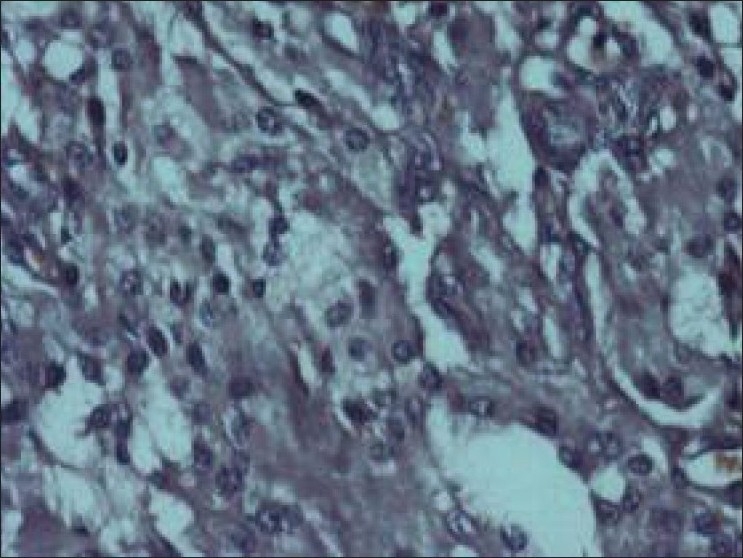
Microscopy of biopsy samples reveal foamy histiocytes and absence of Michaelis Gutmann bodies

After treatment with culture specific antibiotic cystoscopy was done, it revealed approximately 60×50 mm mass protruding from left lateral wall of bladder [Figure 2]. Complete endoscopic resection was done. Histological examination of specimen revealed chronic inflammatory xanthogranulomatous cystitis. Xanthogranulatous macrophages was negative for Periodic acid schiff (PAS) positive material and calcospherules (Michaelis-Guttman bodies) [Figure 3]. Staining for AFB was negative. Immunohistochemical staining for cytokeratin was also found to be negative.

Postoperative course was uneventful. The patient received antibiotics for four weeks. At follow-up after three months, he was asymptomatic and urine culture was sterile.

## DISCUSSION

XC is a rare benign chronic inflammatory disease and majority of the reported cases are associated with urachal diverticula.[5] It was first described in the medical literature in 1932 by Wassiljew.[4] The disease does not have a specific clinical findings other than lower abdominal pain and cystitis like symptoms, umbilical discharge and occasional hematuria. The etiology of XC is unknown. A number of theories are there regarding its origin as immunological disorders,[6,7] abnormal lipid metabolism,[8] metaplasia of urothelium due to chronic infection.[9]

Since medical treatment is ineffective, conservative management is rarely employed.[10] The curative treatment of choice is surgical resection.[6,10] Localized disease may be amenable to simple tumour excision. However, when disease is combined with urachal remnant or adenoma, partial cystectomy is preferred.[10] Additional Chronic suppressive antibiotic therapy and urinary astringents may be helpful. Routine excision of isolated XC lesion may not be indicated.[6]
